# COVID-19 and Cardiac Arrhythmias: Lesson Learned and Dilemmas

**DOI:** 10.3390/jcm13237259

**Published:** 2024-11-29

**Authors:** Federico Blasi, Marco Vicenzi, Roberto De Ponti

**Affiliations:** 1 Department of Science and High Technology, University of Insubria, 21100 Varese, Italy; federico.blasi.md@gmail.com; 2 Cardiology Unit, Department of Internal Medicine, Ospedale di Circolo, ASST-Rhodense, 20017 Rho, Italy; 3 Department of Clinical Sciences and Community Health, University of Milan, 20122 Milan, Italy; marco.vicenzi@unimi.it; 4 Cardiology Unit, Department of Cardiothoracic and Vascular Area, Fondazione IRCCS Ca’ Granda Ospedale Maggiore Policlinico, 20122 Milan, Italy; 5 Department of Medicine and Surgery, University of Insubria, 21100 Varese, Italy

**Keywords:** cardiac arrhythmias, COVID-19, SARS-CoV-2 infection, bradyarrhythmia, tachyarrhythmias, oral anticoagulation

## Abstract

Over the last few years, COVID-19 has attracted medical attention both in terms of healthcare system reorganization and research. Among the different cardiovascular complications of the SARS-CoV-2 infection, cardiac arrhythmias represent an important clinical manifestation requiring proper therapy both in the acute and post-acute phase. The multiparametric in-hospital monitoring of COVID-19 patients frequently detects new-onset or recurrent cardiac arrhythmias. As many patients are monitored remotely from cardiology departments, this setting calls for proper arrhythmia interpretation and management, especially in critically ill patients in the intensive care unit. From this perspective, the possible pathophysiologic mechanisms and the main clinical manifestations of brady- and tachyarrhythmias in COVID-19 patients are briefly presented. The progressively increasing body of evidence on pathophysiology helps to identify the reversible causes of arrhythmias, better clarify the setting in which they occur, and establish their impact on prognosis, which are of paramount importance to orient decision making. Despite the accumulating knowledge on this disease, some dilemmas in the management of these patients may remain, such as the need to implant in the acute or post-acute phase a permanent pacemaker or cardioverter/defibrillation in patients presenting with brady- or tachyarrhythmias and lifelong oral anticoagulation in new-onset atrial fibrillation detected during SARS-CoV-2 infection.

## 1. Introduction

Cardiac arrhythmias are a known complication of SARS-CoV-2 infection and are even more common in critically ill COVID-19 patients [1]. In fact, the occurrence of cardiac arrhythmias in patients hospitalized for COVID-19 was initially reported in retrospective studies from Wuhan, China; in one study, 16.7% of patients experienced some kind of arrhythmia, with incidences up to 44.4% in patients admitted to the intensive care unit (ICU) [2]. Moreover, palpitations were reported as the first symptom of the infection in 7.3% of patients in another case series [3]. Finally, a survey by the Heart Rhythm Society, including 1197 patients, revealed tachyarrhythmia to be the most common rhythm disorder in COVID-19 patients (21%), whereas life-threatening arrhythmias, such as ventricular tachycardia (VT) or ventricular fibrillation (VF), were reported in 4.8% of the cases [4].

Both cardiac and extracardiac factors can contribute to the development of cardiac arrhythmias in COVID-19 patients. These factors include the overexpression of angiotensin II (AT2), direct viral damage to lung and heart tissues leading to myocarditis, myocardial dysfunction, and acute respiratory distress syndrome, which can eventually result in myocardial hypoxia and subsequently lead to cardiac arrhythmias. Additionally, abnormal immune responses often seen in COVID-19 patients, along with myocardial stress caused by pulmonary hypertension, electrolyte or volume imbalances, and myocardial ischemia, can also serve as common triggers for cardiac arrhythmias in these patients [1]. Cardiac injury in patients with COVID-19 infection is associated with critical or fatal courses of the disease and a higher probability of admission to the ICU [5]. Moreover, in the presence of myocardial involvement and a severe course of COVID-19, the incidence of cardiac arrhythmias is estimated to be up to 30.1% versus 2.8% in mild forms of the disease [6]. 

While knowledge of the pathophysiology of this disease has been progressively accumulated, reports of supraventricular and ventricular arrhythmias specifically associated with COVID-19 have shown a wide spectrum of brady- and tachyarrhythmias occurring in these patients [4]. In this population, other than therapies for the SARS-CoV-2 infection, specific antiarrhythmic treatments are necessary in several cases for correct patient management, although a gap in the evidence exists about the proper timing and type of therapy, especially when dealing with a device implant. This paper, starting from the pathophysiologic mechanisms that may lead to the development of cardiac arrhythmias during SARS-CoV-2 infection, will briefly present the spectrum of cardiac arrhythmia reported in the recent literature. A perspective on the possible therapeutic options and the gray zones in the management of these patients will also be given.

## 2. Pathophysiologic Mechanisms of Cardiac Arrhythmias During SARS-CoV-2 Infection

**Figure 1:**
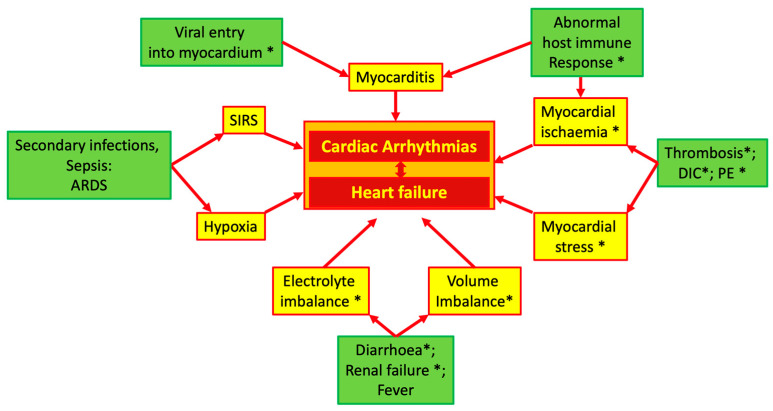
Possible mechanisms of cardiac arrhythmias in COVID-19. Asterisk indicates the influence of ACE system imbalance. Abbreviations: ACE: angiotensin converting enzyme; ARDS: acute respiratory distress syndrome; DIC: diffuse intravascular coagulation; PE: pulmonary embolism; SIRS: systemic inflammatory response syndrome.

In COVID-19 patients, multiple pathophysiologic mechanisms may generate cardiac arrhythmias. Figure 1 summarizes these mechanisms, which will be briefly discussed in the next paragraphs of this section.

### 2.1. Proarrhythmogenic Effects of AT2

The angiotensin converting enzyme 2 (ACE2) is a trans-membrane glycoprotein categorized within the dipeptidyl carboxypeptidase family and represents the entry point for SARS-CoV-2 into human cells [7]. This interaction between ACE2 and the SARS-CoV-2 spike protein leads to the internalization of the ACE2 receptor in the cell and its downregulation, significantly reducing its activity on the cell membrane’s outer surface [8]. This process subsequently reduces the deactivation of AT2 and decreases the production of angiotensin (1–7). AT2 is a potent vasoconstrictor that stimulates the synthesis of aldosterone and increases the sympathetic tone, promoting endothelial dysfunction, hypercoagulability, and oxidative stress [7]. On the other hand, angiotensin (1–7) exerts a protective action on the cardiovascular system, promoting vasodilation and inhibition of the AT2 pathway; in addition, it increases cardiac output and restores endothelial function [7,9]. 

The proarrhythmogenic effects of AT2 are summarized in Table 1. Renin–angiotensin system (RAS) activation is already known to predispose patients with heart failure to reentrant arrhythmias by reducing cell coupling and decreasing conduction velocities [10]. Increased levels of AT2 can lead to refractoriness dispersion, reduced action potential duration, induction of early and delayed afterdepolarizations, and electrical uncoupling [11,12,13]. All these effects were shown to be reversible with the administration of the AT1R blocker losartan [13]. AT2 also stimulates norepinephrine production and its release from the presynaptic nerve endings, promoting the expression of the endothelin-1 gene in endothelial cells, which exerts its own proarrhythmic effect on the heart [14,15,16]. Excessive catecholamine release induced by AT2 can stimulate foci rich in sympathetic nerve endings, potentially triggering atrial arrhythmias. As an example, atrial fibrillation (AF) is already known to be associated with the downregulation of AT1R and the upregulation of AT2R [17]. This effect may be intensified by concurrent hypoxia, as demonstrated by Maruyama et al., who showed how, in anoxic conditions, there is a release of endogenous norepinephrine in the myocardium [18]. Furthermore, AT2 has been found to act in the area postrema in the central nervous system, resulting in the central inhibition of the cardiac parasympathetic tone [19]. This autonomic imbalance can lead to the loss of heart rate variability and increase the risk of malignant VA [10]. 

Of note, there is also a high density of AT1 receptors in the cardiac conduction system [20], and the activation of AT1R by AT2 in the sinoatrial and atrioventricular nodes and Purkinje fibers triggers various intracellular responses that can lead to spontaneous electrical activity and reduced intracellular resistance, and significantly increase conduction velocity [21,22,23,24]. 

The vasoconstrictive effect of AT2 is particularly pronounced in the coronary circulation, and high levels of circulating AT2 can induce focal myocardial necrosis, which may become a source of abnormal electrical activity [25,26]. As shown in Figure 1, in addition to the direct effects, an unbalanced ACE system can indirectly cause or facilitate cardiac damage and, thus, induce arrhythmias due to its specific properties in regulating euvolemia and its effects on the coagulation system.

### 2.2. Hypoxia

Hypoxia caused by acute respiratory failure in COVID-19 patients results in damage to cardiomyocytes through a mechanism akin to what is observed in patients experiencing acute myocardial infarction [27]. The lowering of intracellular pH and the increase in the cytosolic calcium concentration can cause early and late depolarization [28]. Additionally, hypoxia raises the concentration of extracellular potassium, reducing the thresholds for depolarization and thereby increasing the risk of arrhythmias [27].

### 2.3. Abnormal Inflammatory Response

The progression of COVID-19 is typically described in three stages. The first stage represents the early phase of infection, during which typical COVID-19 symptoms manifest. In the second stage, the virus invades and replicates within pneumocytes, setting off an inflammatory immune response that can sometimes result in acute respiratory distress syndrome. For some patients, the disease advances to the third stage, characterized by a hyperinflammatory state, wherein a cytokine storm triggers dysfunction in multiple organs [29]. Among the cytokines implicated in immune cell-mediated myocardial injury, there is interleukin-6, tumor necrosis factor-α, and interleukin-1 [30]. These inflammatory cytokines can overstimulate the cardiac sympathetic system, exacerbating preexisting coronary atherosclerosis [31], and can directly affect the function of ion channels in cardiomyocytes, leading to a prolonged action potential duration and potentially causing QT interval prolongation [32,33]. Finally, in COVID-19, systemic inflammation could trigger life-threatening arrhythmic events in individuals with preexisting long-QT syndrome [33]. 

### 2.4. Myocardial Ischemia

COVID-19 infection has also been associated with the occurrence of acute myocardial infarction. This may occur due to the activation of inflammatory cells within preexisting atherosclerotic plaques, potentially leading to plaque rupture [34]. Cytokines like interleukin-6 and tumor necrosis factor-alpha, released during the hyperinflammatory stage, can create a prothrombotic environment, contributing to microvascular dysfunction [35]. Another potential cause of myocardial ischemia is infection-induced vasculitis, either through direct viral entry into myocardial vessel endothelial cells or through an indirect immunological response [36].

### 2.5. Myocardial Inflammation

Myocarditis has been documented in COVID-19 patients in previous studies, and various hypotheses have been proposed to explain its pathophysiology [37]. One hypothesis suggests that the virus directly penetrates myocardial cells by utilizing the viral spike protein, which binds to the ACE-2 receptor on the myocardial cell membrane, leading to direct myocardial injury [27]. This spike protein binding can downregulate the ACE-2 receptor, potentially resulting in the accumulation of AT2 and adverse remodeling of the myocardium, which may disrupt electrical conduction and increase the risk of arrhythmias [38]. Another mechanism involves cellular-based inflammation, with CD8+ T-lymphocytes causing myocyte inflammation as part of systemic inflammation. This process is amplified by a significant cytokine release, intensifying T-lymphocyte activity and further cytokine release, creating a vicious cycle [37]. Additionally, a recent study demonstrated decreased levels of CD14dimCD16+ monocytes in the peripheral blood of COVID-19 patients with severe coronary artery disease [39]. These monocytes are responsible for cytokine release and favor systemic inflammation. The low levels in the peripheral blood may be caused by their migration into the heart and lungs, where their hyperactivation promotes tissue damage [39]. In acute inflammatory phases of COVID-19 myocarditis, cardiac arrhythmias may arise due to gap junction dysfunction, electrophysiological remodeling, abnormal calcium handling, and downregulation of potassium channels, leading to prolonged repolarization [40]. Cardiac arrhythmias can also persist in the post-acute phase of COVID-19, possibly due to residual myocardial scarring facilitating reentrant arrhythmias [41].

### 2.6. Electrolyte Imbalance

Electrolyte imbalance and its impact on preexisting arrhythmias have been extensively studied. The importance of different sensitivities to K^+^ among different types of myocardial fibers and the modification of the electrophysiologic effects of K^+^ by the Ca^2+^ and Na^+^ levels are well known [42]. In a case series, electrolyte imbalances were reported in 7.2% of 416 hospitalized COVID-19 patients [43]. These imbalances were attributed to COVID-19-associated diarrhea or renal injury, among other causes. A retrospective analysis by Diao et al. [44] found that acute kidney injury occurred in 27% of 85 COVID-19 patients hospitalized with the infection, thus facilitating electrolyte imbalance, such as hypokalemia, hypomagnesemia, and hypophosphatemia. In a retrospective study [45], acid–base alterations were found in 79.7% of the COVID-19 patients, with metabolic alkalosis (33.6%) as the most frequent alteration, followed by respiratory alkalosis (30.3%) and combined alkalosis (9.4%). Importantly, the acid–base disorders are associated with hypokalemia and Cl^−^ imbalance [46]; it has been reported that the correction of hypokalemia in these patients was difficult due to continuous renal potassium loss resulting from a disordered RAS [46]. Finally, in a cohort study, 18% of the COVID-19 patients presented severe hypokalemia (plasma potassium < 3 mmol/L), and 38% had hypokalemia (plasma potassium 3–3.5 mmol/L) [47].

### 2.7. Drug Side Effects

Regarding QT-prolonging drugs, such as hydroxychloroquine, chloroquine, or azithromycin with or without hydroxychloroquine, they were previously identified in several reviews as potential causes of cardiac arrhythmias in the early stages of the pandemia due to their ability to prolong the QT interval [27,48,49]. However, since these medications are no longer recommended for COVID-19 treatment, their role in causing arrhythmias is now negligible. Dexamethasone and remdesivir have been found to benefit COVID-19 patients without affecting the QT interval duration [50], but remdesivir has been associated with transient sinus bradycardia and with return to a normal sinus rhythm after drug discontinuation [51,52]. Importantly, a meta-analysis reported that sinus bradycardia related to remdesivir administration is not associated with increased mortality [53].

## 3. Clinical Manifestations

Considering the multiple underlying mechanisms in arrhythmogenesis, the variable clinical presentation of SARS-CoV-2 infection, and the possible comorbidities, the rhythm disorders described in COVID-19 patients can be multiple, their impact on the patient outcomes variable, and the short- and long-term treatments different according to the clinical presentation. Table 2 summarizes the most frequently reported arrhythmias observed in COVID-19 patients. 

**Figure 2:**
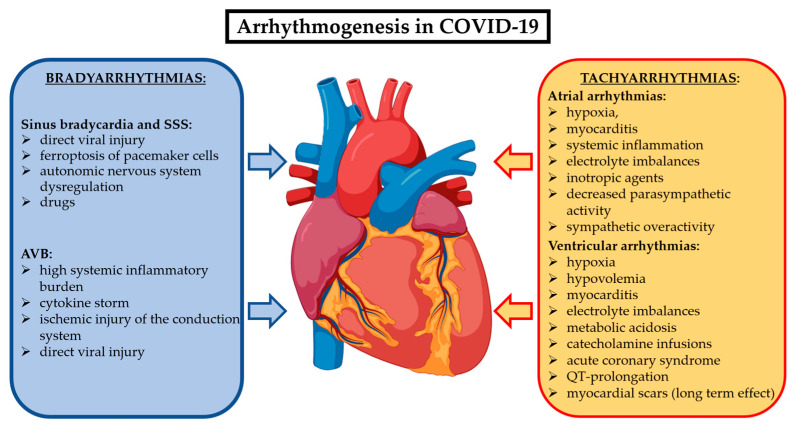
Most accredited mechanisms involved in COVID-19 to generate bradyarrhythmias and supraventricular and ventricular tachyarrhythmias. Abbreviations: AVB: atrioventricular block; SSS: sick sinus syndrome.

Cardiac arrhythmias related to COVID-19 could occur as new-onset or recurrent episodes during the acute phase and, in some cases, they may persist in the post-acute phase beyond three months, configuring the long COVID syndrome. There are multiple mechanisms underlying the arrhythmogenesis of the different brady- and tachyarrhythmias in COVID-19 patients. They are summarized in Figure 2. While the data on cardiac arrhythmias during the acute phase of COVID-19 are abundant, the evidence on their prevalence and impact on the long COVID syndrome are limited.

### 3.1. Bradyarrhythmias

Although the current definition of sinus bradycardia is a sinus rate < 60 beats per minute, in healthy individuals of any age, a sinus rate below this threshold can be indicative of a physiological response to a parasympathetic influence related to training or sleeping. Therefore, as recommended by the international guidelines for any case of bradyarrhythmia [54] and in the case of a COVID-19 patient presenting with sinus bradycardia, the evaluation should not be limited to the mere consideration of the sinus rate but should also include a complete history of the patient to assess the previous cardiovascular status, the presence of symptoms unambiguously related to bradycardia, and the possible relationship with the occurrence of the SARS-CoV-2 infection. This clinical evaluation should be routinely performed in each case to avoid an overdiagnosis of bradyarrhythmias possibly resulting in unnecessary pacemaker implantation. This is of key importance considering that data from a global survey [55] reporting a higher complication rate (13.9% at 30 days) in device implantation in patients with active COVID-19 infection with a fatal outcome directly related to device implant.

**Figure 3:**
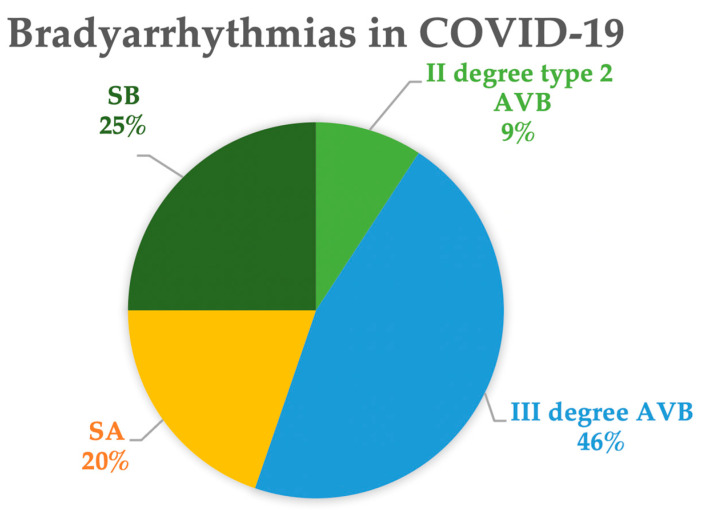
Types of bradyarrhythmia in COVID-19. Data are extracted from references [57,58,59,60,61,62,63,64,65,66,67,68,69,70,71,72,73,74,75,76,77,78,79,80,81,82,83,84,85,86,87,88,89,90,91,92,93,94,95,96,97,98,99,100,101,102]. Abbreviations: AVB: atrioventricular block; SA: sinus arrest; SB: sinus bradycardia.

Both sick sinus syndrome (SSS) and various degrees of atrioventricular block (AVB) have been described in the literature [56]. Figure 3 shows the proportions of the different types of bradycardias extracted from the literature [57,58,59,60,61,62,63,64,65,66,67,68,69,70,71,72,73,74,75,76,77,78,79,80,81,82,83,84,85,86,87,88,89,90,91,92,93,94,95,96,97,98,99,100,101,102]. Among these cases, 45% exhibit bradycardias associated with sinus node dysfunction, including marked or relative sinus bradycardia (25%) or sinus arrest (20%). In the remaining cases, complete AVB was observed in 46% and second-degree type 2 AVB in 9% of the patients.

Han et al. demonstrated that sinoatrial node damage caused by the direct infection of SARS-CoV-2 into pacemaker cells is a potential mechanism of COVID-19-induced bradycardia [103]. The viral infection leads to ferroptosis, and eventually, cell death in pacemaker cells. Thus, patients with damage to the sinoatrial node can suffer from bradycardia even in the post-acute or chronic phases, explaining a part of the relevant mechanism of heart rate dysregulation in long COVID [104].

Possible mechanisms of complete AVB include a high systemic inflammatory burden and cytokine storm causing ischemic injury to myocardial cells and affecting the intrinsic conduction system [105]. Another possible mechanism involves a direct viral injury to the myocardium and the conduction system itself manifesting as a transient complete heart block [106]. Dagher et al. described four cases of COVID-19 patients who developed a transient high-degree AVB during their hospitalization, none of which required permanent pacing, thus suggesting a transient condition, linked to an inflammatory response [75].

Even if the conduction disturbance could only be transient, it may still be a sign of a severe form of the disease. In fact, Chinitz et al. [59] reported seven cases of bradyarrhythmia in patients without evidence of left ventricular systolic dysfunction or acute cardiac injury but with elevated inflammatory markers. Symptomatic persistent high-degree AVB was present at initial presentation in 43% of patients, while the remaining developed sinus arrest or paroxysmal high-degree AVB during hospitalization. The rhythm complications were not secondary to severe forms of respiratory disease, as bradyarrhythmias occurred before the respiratory symptoms. Due to the perceived life-threatening condition, 71% of patients received permanent pacemakers, while in two cases, the pacing was only temporary. Among these patients, 57% died from COVID-19-related complications during the initial hospitalization, and this proportion increased to 71% at 3 months. Thus, these authors suggest that conduction disorders could be a potential marker of a poor prognosis. 

### 3.2. Tachyarrhythmias

As shown in Table 1, both supraventricular and ventricular tachyarrhythmias have been described in association with COVID-19. SARS-CoV-2 infection can represent a transient reversible trigger for tachyarrhythmias or can simply unveil a preexisting substrate. To understand which of the two conditions occurs is of paramount importance to select the proper treatment for the individual patient. 

The most common and challenging tachyarrhythmias will be presented in the next sections. 

#### 3.2.1. Inappropriate Sinus Tachycardia

The occurrence of inappropriate sinus tachycardia (IST) is a frequent finding in COVID-19 patients [107]. Although sinus tachycardia is commonly observed in the acute phase and clearly related to the inflammatory state and hypoxia, IST is defined as an elevated resting heart rate (>100 bpm) clearly of sinus origin with an exaggerated response to physical or emotional stress. After the acute infection phase, IST can persist in the weeks or months after, being responsible for symptoms such as fatigue and shortness of breath, which can result in functional limitations. IST was observed in 20% of patients presenting with post-COVID-19 syndrome [107] and was associated with a reduction in various heart rate variability parameters, particularly those linked to cardiovagal function. The underlying cause of this phenomenon may be an imbalance in the cardiac autonomic nervous system, characterized by decreased parasympathetic activity related to the SARS-CoV-2 infection [108,109]. For this reason, patients presenting with IST are expected to benefit from beta-blockers with a daily dose titrated based on symptom relief and individual tolerance.

#### 3.2.2. Atrial Fibrillation

Several factors, including hypoxia, myocarditis, systemic inflammation, electrolyte imbalances, inotropic agents, and sympathetic nervous system overactivity, can contribute to AF in COVID-19 patients [110]. AF is the most common heart rhythm disorder in these patients [4]. In patients with a history of paroxysmal AF, there is a recurrence in 23% to 33% of patients with sepsis or acute respiratory distress syndrome caused by COVID-19. Among those with no history of the arrhythmia, new-onset AF was diagnosed in 8% of patients hospitalized for COVID-19 and was associated with a worse outcome [111]. The hypothesis that the occurrence of AF or atrial flutter is not specific to COVID-19 but is a response to systemic inflammation is supported by the observation that the incidence of atrial arrhythmias was similar in patients hospitalized for COVID-19 compared to those with influenza (13% versus 12%, respectively) with, however, a significant lower mortality rate in the latter group [112]. Interestingly, also, in this series, the occurrence of new-onset AF, observed in 4% of the cases was associated with a significantly higher mortality compared to patients with a prior history of AF (55 versus 24%, respectively, *p* = 0.01). New-onset AF occurred in older patients with significantly higher levels of inflammatory markers, troponin, and brain natriuretic peptide [112].

In the acute phase, treatment options depend on hemodynamic stability, with synchronized cardioversion for unstable patients and beta-blockers or calcium channel blockers as first-line therapy for rate control in stable patients [49,113]. Other options include digoxin/digitoxin and amiodarone for sinus rhythm restoration, with careful monitoring of its QT-prolonging side effect [49]. Additionally, addressing underlying triggers like hypoxia, electrolyte imbalances, acidosis, and volemic status is crucial [49,113]. Anticoagulation after the detection of AF is recommended in all patients with sepsis or heart failure [28], but the evidence about post-recovery anticoagulation is less clear and will be discussed later in this article. 

**Figure 4:**
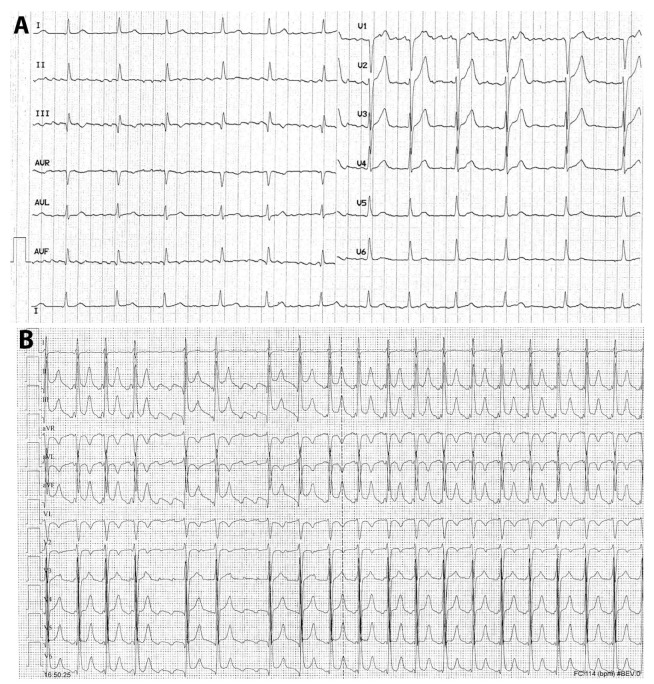
(**A**,**B**) Twelve-lead electrocardiogram of a case with atrial fibrillation (**A**) and typical atrial flutter (**B**). In (**A**), the absence of distinct repeating P-waves with irregular atrial activation, which are diagnostic criteria for atrial fibrillation, is evident; the ventricular response is modulated by pharmacologic agents acting on the atrioventricular conduction. In (**B**), P-waves with sawtooth morphology (negative in the inferior and left precordial leads and positive in aVL and V1) is clearly observed in the right-hand side; importantly, during the increased sympathetic tone, possibly during infections, the atrioventricular conduction ratio is frequently 2:1 with a ventricular response of 140–150 bpm, as in the right-hand side of this figure. In both arrhythmias, 12-lead electrocardiographic recording is essential for a correct diagnosis.

Although in the literature AF and atrial flutter are grouped together as if they were the same arrhythmia, they differ in terms of pathophysiology, electrocardiographic pattern, clinical presentation, and therapy (Figure 4A,B). Therefore, typical atrial flutter, being sustained by an anatomic and well-defined right atrial re-entry, is prone to recur even after the acute phase of COVID-19. Consequently, elective ablation of the arrhythmia circuit may be considered if this arrhythmia has been documented. 

#### 3.2.3. Ventricular Arrhythmias

Life-threatening ventricular arrhythmias, namely VT or VF, are reported in 5.9% of patients hospitalized for COVID-19 [114]. Cardiac injury, defined by elevated troponin plasma levels, was found in 27.8% of the patients, and it was associated with cardiac dysfunction and arrhythmias. In fact, elevation of the troponin levels was significantly associated with an increased rate of malignant ventricular arrhythmias (17.3 versus 1.5%, *p* < 0.001), a higher use of glucocorticoid therapy, and mechanical ventilation [114].

Management is similar to all patients with malignant ventricular arrhythmias and involves defibrillation for VF and synchronized electrical cardioversion for hemodynamically unstable VT [115,116]. Intravenous amiodarone and beta-blockers are used for recurrent VT or electrical storm, and sedation and intubation can become necessary in case of refractory arrhythmias [115]. Correcting underlying reversible triggers such as hypoxia, hypovolemia, electrolyte imbalances, and metabolic acidosis is essential. If ventricular arrhythmia is suspected to be secondary to an acute coronary syndrome, invasive coronary angiography should be considered and promptly performed [115]. In case of QT-prolongation and torsade de pointes, all QT-prolonging drugs should be withdrawn immediately, while intravenous magnesium and the correction of potassium levels are indicated. If necessary, isoproterenol or temporary pacing can be used [115,116]. Invasive treatment of life-threatening arrhythmias should be performed in a heart center with circulation support devices [115,116].

## 4. Therapeutic Dilemmas in the Management of Arrhythmias Associated with COVID-19

There are still gaps in the evidence regarding the treatment of patients with COVID-19 and cardiac arrhythmias, especially those in critical conditions. This is crucial for clinicians in everyday practice, as the choice of the appropriate therapy could be difficult. While the clinicians should refer to the international guidelines [54,113,116] for the treatment of supraventricular and ventricular arrhythmias, the presence of an inflammatory state and ongoing infection, as well as the presence of a preexisting cardiovascular disease and comorbidities, should be carefully considered for proper patient management. As mentioned before, in the management of these patients, the main challenge is the discrimination between arrhythmias due to reversible causes and arrhythmias related to a silent preexisting substrate unveiled by a SARS-CoV-2 infection. In fact, while the first are treated by reverting the casual alterations, the second require careful evaluation, as they may recur during follow-up. On the other hand, SARS-CoV-2 infection can produce a de novo and permanent cardiac damage, resulting in the creation of an arrhythmogenic substrate. In this light, whether to implant a permanent pacemaker or an internal cardioverter/defibrillator (ICD) in patients presenting with brady- or life-threatening tachyarrhythmias represents a clinical dilemma, as well as to prescribe lifelong oral anticoagulation in patients with new-onset AF at risk for thromboembolic events. In the next sections, these clinical dilemmas will be briefly addressed and discussed.

### 4.1. Need for Permanent Pacing in Bradyarrhythmias

As shown by case series [56,59,74,76], bradyarrhythmia during SARS-CoV-2 infections may be reversible following the acute phase of the illness. From a preliminary analysis of the literature [57,58,59,60,61,62,63,64,65,66,67,68,69,70,71,72,73,74,75,76,77,78,79,80,81,82,83,84,85,86,87,88,89,90,91,92,93,94,95,96,97,98,99,100,101,102], SSS and AVB seem reversible in roughly half of the cases. Interestingly, sinus node dysfunction seems reversible in two-thirds of the cases, and less severe atrioventricular conduction disturbances, such as second-degree type 2 AVB, occur transiently in almost 90% of the cases. Conversely, in patients with complete AVB, the conduction disturbance persists in two-thirds of the cases and requires permanent pacing. As mentioned before, it should be considered that remdesivir administration is associated with reversible sinus bradycardia, observed in 22.3% of patients receiving this drug versus 9.8% of controls [51]. 

Currently, there is no solid evidence or clear indication in the literature regarding which cases should undergo permanent pacemaker implantation. In symptomatic patients with hemodynamic impairment related to bradyarrhythmia, initial therapy with temporary intracavitary pacing until resolution of the acute phase of the infection could be an option to avoid unnecessary implants. This prevents possible perioperative complications, higher in COVID-19 patients [55], and allows for distancing from the acute phase, during which potential bacterial superinfections could lead to short- or long-term infective complications of the device. This is acceptable, provided that the time of temporary pacing is not too prolonged, as it could represent a source of infective complications by itself. 

Once the initial phase is overcome and sinus node dysfunction or advanced AVB persists, it seems necessary to proceed to permanent pacemaker implantation. However, as mentioned before, the main challenge is represented by the evaluation of patients in whom SSS and AVB have resolved but might recur, since, in some cases, the SARV-CoV-2 infection can be a concurring cause in unveiling preexisting silent rhythm disturbances. In these cases, wise and shared decision making should be performed, mainly based on clinical patients’ characteristics, including age, family history, history of unexplained syncope, preexisting PR interval prolongation and/or bundle branch block, and comorbidities known to be associated with SSS or paroxysmal AVB.

### 4.2. Need for ICD Implant

In cases of ventricular arrhythmias during the acute phase, an evaluation for structural heart disease or potential reversible triggers should be performed [115]. According to the current guidelines of the European Society of Cardiology, when possible, patients should be categorized according to the etiology of ventricular arrhythmia [116]. Therefore, when a secondary and correctable factor is clearly identified as the determinant of the arrhythmia occurrence, such as electrolyte imbalances, acute myocardial ischemia, drug adverse effects, or other reversible causes, ICD implantation is not indicated [116]. In some cases, because of the complexity of the clinical presentation, this discrimination may not be easy. Cardiac magnetic resonance, whenever possible, can better characterize left ventricular dysfunction and the presence of the arrhythmogenic substrate [117]. The presence of late gadolinium enhancement could be the substrate related to a preexisting cardiomyopathy or to the sequalae of SARS-CoV-2 myocarditis, which shows the patients at risk of future recurrences of ventricular arrhythmia. On the other hand, intramyocardial edema without scarring could still be related to the acute inflammatory phase, which may represent a reversible cause of arrhythmia and ventricular dysfunction, if resolved with no sequelae. The short-term use of the wearable defibrillator allows more time for final decision making in cases of uncertain reversibility of cardiac involvement causing ventricular arrhythmia [48]. In fact, a recent case report of post-COVID-19 fast VTs in a young female patient represents a proof of concept that the wearable defibrillator can be taken into account in the shared decision making process between the healthcare provider and the patient, which allows for a safe discharge home while the self-limiting course of the disease is continuously monitored [118].

### 4.3. Need for Lifelong Oral Anticoagulation in New-Onset AF During COVID-19

While anticoagulation is a common therapy in severe forms of COVID-19 during the acute phase, in the presence of new-onset AF, which may be secondary to transient systemic inflammation, its long-term benefit in the prevention of thromboembolic events in patients at increased risk (CHA_2_DS_2_-VASC score ≥ 2) is less clear. 

Patients with AF and reversible triggers have been excluded from clinical trials on oral anticoagulants (OACs) [119,120], thus resulting in a lack of evidence from randomized, controlled trials about the risk–benefit profile of long-term OAC in patients with new-onset AF during sepsis. In fact, the use of OAC in the so-called secondary forms of AF is uncommon in daily clinical practice in patients surviving hospitalization with sepsis and new-onset AF. A recent large multicenter cohort study confirmed that the use of OAC after hospitalization for sepsis is uncommon, despite increasing over time, from 18% in 2011 to 24% in 2017 [121]. On the other hand, the incidence of recurrence after new-onset AF in patients with sepsis is high (approximately 40% at 1 year), suggesting a possible benefit of long-term OAC [122]. The recent 2024 European Guidelines for AF addressed the topic of trigger-induced AF defined as new AF in the immediate association of a precipitating and potentially reversible factor [113]. According to retrospective and observational data, patients with trigger-induced AF seem to have the same thromboembolic risk as patients with primary AF, and thus, long-term OAC should be considered (class of recommendation: II A, level of evidence: C) in suitable patients with trigger-induced AF at increased thromboembolic risk to prevent ischemic stroke and systemic thromboembolism, as calculated with the new CHA_2_DS_2_-VA score [113]. Therefore, the final decision for a long-term OAC of suitable patients after a new-onset AF during COVID-19 should be shared with the patient and the family, based on individual patient characteristics and the risk/benefit ratio.

## 5. Long COVID and Cardiac Arrhythmias

Currently, the term long COVID refers to the persistence of symptoms beyond 3 months of SARS-CoV-2 infection and lasting for at least 2 months that are not explained by any other illness. In addition to the cardiovascular systems, multiple organs can be involved, such as lungs, kidney, brain, skeletal muscles, and endocrine glands, with possible multifaceted clinical manifestations, which may resemble the patterns observed in past epidemics and infections [123]. Long COVID syndrome is considered a vacillating disease, characterized by a diverse range of symptoms with multiple factors contributing to the variability in prevalence estimates, such as preexisting diseases, variable sociodemographic conditions, and variable sample size and methodologies used in studies [124]. 

There is paucity of data specifically on cardiac arrhythmias in the long COVID syndrome. In a recent metanalysis [125], an increased occurrence of presenting with sinus bradycardia or tachycardia, supraventricular or ventricular arrhythmias was found in long COVID patients compared to controls, although with substantial heterogeneity. Persistently elevated sinus heart rates were observed in a study using wearable devices with substantial intraindividual variability, possibly related to different levels of autonomic nervous system dysfunction or persistent inflammation after SARS-CoV-2 infection [126]. The impact of SARS-CoV-2 infection on the autonomic nervous system influencing the cardiovascular one is also supported by the finding of new-onset and persistent postural orthostatic tachycardia syndrome (POTS) in post-COVID patients with elevated inflammatory or autoimmune markers, while neurogenic syncope or orthostatic hypotension can be the other manifestations [127]. As POTS and other symptoms of new-onset autonomic dysfunction can be debilitating for the patients, their treatment represents a main concern for the clinicians. While the current specific guidelines are lacking as specific clinical data are missing [128,129], increased sodium chloride and fluid intake, waist-high compression stockings, and sitting or supine exercise are usually advised as non-pharmacologic measures in these patients. In refractory patients, betablockers, fludrocortisone, midodrine, and ivabradine have been used with inconsistent but occasionally positive results on symptoms [130]. 

## 6. COVID-19, Cardiac Arrhythmias, and New Opportunities

As discussed so far, the SARS-CoV-2 infection may cause new-onset cardiac arrhythmias. In the general population, the lockdown had a strong impact on the new diagnosis of arrhythmias, such as AF, resulting in notable reductions and limiting access to timely care, which is crucial to prevent stroke and other complications [131]. On the other hand, the COVID-19 pandemic created opportunities to implement telemedicine options, which necessarily appeared as the most appropriate methodology to evaluate patients both affected and unaffected by the virus. While remote monitoring (RM) represented an opportunity to limit viral diffusion during the pandemic, currently, the global adoption of this methodology for patient care can represent a shift in paradigm in the diagnosis and treatment of patients affected by cardiac arrhythmias. In fact, despite the promises of RM, this technology has remained underutilized for decades for several reasons, such as lack of dedicated personnel, organizational model, and reimbursement [132]. The COVID-19 pandemic forced a new era of telehealth and virtual solutions; the use of RM with different devices demonstrated to be a safe alternative to in-person visits with enhanced patient satisfaction and improved clinical outcomes [133]. Specifically, RM for cardiac implantable electronic devices (CIEDs), such as loop recorders, pacemakers, ICD, and devices for cardiac resynchronization therapy, not only efficiently replaces the in-office follow-up visits but also allows for a greater continuity in the patient follow-up with timely intervention whenever needed [134]. Importantly, according to an Italian survey [135], the COVID-19 pandemic caused an acceleration in the use of RM of CIEDs in patients with cardiac arrhythmias with a significant increase in 72% of the participating centers. Moreover, data obtained by the RM of CIEDs showed a negative impact of the COVD-19 pandemic and lockdown restrictions in monitored patients with a significant increase in both supraventricular and ventricular arrhythmias associated with a decrease in physical activity and heart rate variability, independent from SARS-CoV-2 infection prevalence [136]. Finally, in patients with heart failure and reduced ejection fraction implanted with cardiac resynchronization therapy devices, RM detected a significant drop in the median activity level during the lockdown and, of note, generated a higher rate of alerts suggestive of a worsening of the heart failure status [137].

## 7. Conclusions

During SARS-CoV-2 infection, the occurrence of cardiac arrhythmias recognizes variable pathophysiologic mechanisms, which may interact and increase the chance of arrhythmic manifestations. This may be associated with a worse prognosis, especially in critically ill patients with new-onset cardiac arrhythmias. Moreover, peculiarly, the pathophysiologic mechanism may act differently in different patients to originate different arrhythmias. In fact, paradoxically, the SARS-CoV-2 infection can be responsible for two opposite arrhythmic patterns, namely SSS and IST, which can be explained by direct viral damage in the first and by persistent dysregulation of the autonomic nervous system in the latter. Therefore, a deeper understanding of SARS-CoV-2 pathophysiology can lead to better recognition, treatment, and prevention of COVID-19-related arrhythmias. Although treatment in these patients may not substantially differ from other patients, there are still some gray zones that may pose dilemmas to clinicians in their decision making.

## Figures and Tables

**Table 1:** Possible proarrhythmic effects mediated by AT2: targets and mechanisms.

**Cardiac electrical activity**
- Reduced cell coupling - Decreased conduction velocities - Refractoriness dispersion - Reduced action potential duration - Induction of early and delayed afterdepolarizations - Spontaneous electrical activity
**Autonomic nervous system**
- Excessive norepinephrine production - Inhibition of cardiac parasympathetic tone - Loss of heart rate variability
**Coronary vessels**
- Endothelial dysfunction - Excessive vasoconstriction - Endovascular thrombosis - Focal myocardial necrosis

**Table 2:** Cardiac arrhythmias in COVID-19 patients.

**Bradyarrhythmias**
- Sick sinus syndrome - Atrioventricular block: second-degree type 2, 2:1, third-degree
**Tachyarrhythmias**
Supraventricular - Inappropriate sinus tachycardia - Atrial premature beat - Atrial fibrillation - Atrial flutter - Atrial tachycardia
Ventricular - Ventricular premature complexes and non-sustained ventricular tachycardia - Sustained ventricular tachycardia - Polymorphic ventricular tachycardia (torsade de pointes) - Ventricular fibrillation

## Data Availability

The sources of the data presented in this perspective paper are cited in the references.
